# Contrast dose determination using effective diameter in patients of unknown weight for dynamic computed tomography of the upper abdomen: a feasibility study

**DOI:** 10.1007/s12194-025-00995-y

**Published:** 2026-01-19

**Authors:** Masaaki Fukunaga, Shota Ichikawa, Koki Ichijiri, Osamu Ito, Takafumi Moriya, Yuki Yamaguchi

**Affiliations:** 1 Department of Radiological Technology, Kurashiki Central Hospital, 1-1-1 Miwa, Kurashiki, 710-8602 Okayama Japan; 2 Department of Radiological Technology, Graduate School of Health Sciences, Niigata University, 2-746 Asahimachi-dori, Chuo-ku, Niigata, 951-8518 Japan; 3 Institute for Research Administration, Niigata University, 8050 Ikarashi 2-no-cho, Nishi-ku, Niigata, 950-2181 Japan

**Keywords:** Effective diameter, Body weight, Iodine contrast dose

## Abstract

**Purpose:**

The purpose of this study was to validate the accuracy of a method for determining the iodine contrast dose using effective diameter (D_eff_) when performing dynamic computed tomography (CT) scans of the upper abdomen in patients with unknown body weight (BW).

**Methods:**

D_eff_ was measured at the heart, at the right diaphragm upper edge, and at the right pulmonary rib diaphragm levels in the localizer radiograph and axial images. Correlation coefficients between D_eff_ and BW were determined for each cross section.

**Results:**

D_eff_axial_ and BW showed the highest correlation at the right diaphragm upper edge level in men (r_S_ = 0.862) and at the right pulmonary rib diaphragm level in women (r_S_ = 0.890).

**Conclusions:**

The BW estimated from D_eff_ showed a strong correlation with measured BW and may serve as a practical alternative in cases where BW is unknown.

## Introduction

The iodine contrast dose is generally determined by body weight (BW) on computed tomography (CT) scans [1]. Other factors such as body surface area (BSA), lean body mass, and fixed dosing protocols may also influence the selection of contrast volume [2, 3]. The patient’s BW is not always measured before the CT scan, especially in emergency contrast-enhanced CT scans. Patients with unknown BW are at a disadvantage in determining the appropriate iodine contrast dose. If the dose of iodine contrast is too low, the desired enhancement may not be achieved. Therefore, even for patients with unknown BW, the iodine contrast dose must be accurately determined and adjusted to achieve the target enhancement.

Methods for estimating BW from CT images have been previously reported. Geraghty et al. reported on the predictive equations for estimating an individual’s BW from a single abdominal CT image [4]. However, this predictive equation is rarely used because it is limited to the first lumbar vertebra on abdominal CT, and it sets different prediction formulas for men and women, requiring the measurement of many anatomical regions. Ichikawa et al. proposed a deep learning method using chest and abdominal CT scout images to estimate patient BW [5]. However, this method is still in the research phase and not yet ready for clinical use. Fukunaga et al. found that the effective diameter (D_eff_) was strongly correlated with BW and used D_eff_ for CT radiation dose management in patients with unknown BW [6]. D_eff_ is easily calculated by measuring the anteroposterior and lateral diameters on a CT scanner; therefore, it presents a practical option to incorporate into daily clinical practice. However, there are no reports on which slice position is optimal for measuring the D_eff_. The correlation coefficient between BW and D_eff_ has been reported to be higher in chest CT images than in abdominal CT images [6]. Furthermore, in the X-ray images, a heatmap analysis showed an emphasis on the diaphragmatic region to predict BW [7]. Based on these findings, we hypothesized that the slice position near the diaphragm was desirable for developing a BW estimation method using D_eff_ in chest, abdominal, and chest-to-pelvis CT.

The purpose of this study was to validate the accuracy of BW estimation using D_eff_ and to develop a method for determining iodine contrast dose using D_eff_ when performing dynamic CT scans of the upper abdomen in patients with unknown BW.

## Methods

### Patients

This retrospective study was approved by the Institutional Review Board (No.3727). Three patient groups were enrolled: Group A to derive a linear regression equation between actual BW and D_eff_, Group B to assess inter-rater reliability, and Group C to evaluate the contrast effect.

#### Group A: patient group to obtain a linear regression equation between actual BW and D_eff_

We enrolled 245 patients in Group A, who underwent low-dose CT scans for lung cancer screening at Kurashiki Central Hospital Preventive Healthcare Plaza between May 1 and July 1, 2021. Exclusion criteria were as follows: scoliosis (1 man), inability to raise both arms (2 men), the subject is not fully contained within the field of view (FOV) (11 men, 5 women). Consequently, the final study population consisted of 226 patients (175 men and 51 women; mean age: 59.2 [range: 31.1 to 81.6] years; mean BW: 65.2 [range: 40.3 to 107.5] kg; mean height: 166.3 [range: 147.1 to 190.0] cm). This group was selected because their height and BW were measured on the same day as the CT scan. These subject data were used to evaluate the accuracy of the D_eff_ measurement and to develop a linear regression equation between actual BW and D_eff_.

#### Group B: patient group to assess inter-rater reliability for measuring D_eff_

The subjects were 50 men and 50 women, each consecutively selected from individuals undergoing low-dose CT scans for lung cancer screening at Kurashiki Central Hospital between May 1 and July 1, 2021. Exclusion criteria were as follows: BW greater than 75 kg (11 men, 2 women), inability to raise both arms (1 man), and the subject is not fully contained within the FOV (5 women).

After applying these criteria, 38 men remained, with a mean age: 58 [range: 41 to 79] years; mean BW: 62.9 [range: 49.6 to 72.9] kg; mean height: 168.2 [range: 153.9 to 185.1] cm. Similarly, 43 women remained, with a mean age: 64 [range: 31 to 80] years; mean BW: 56.1 [range: 40.3 to 71.5] kg; mean height: 157.6 [range: 147.1 to 166.9] cm.

These exclusions were made to account for potential differences in body habitus between sexes that could influence the D_eff_ measurements. Accordingly, intraclass correlation coefficient (ICC) values for inter-rater reliability were calculated separately for men and women. Individuals with a BW of 75 kg or greater were excluded from Groups B and C.

#### Group C: patient group to evaluate the contrast effect

Fifty patients (36 men and 14 women; mean age: 69 [range: 39 to 87] years; mean BW: 60.2 [range: 41.0 to 75.0] kg; mean height: 162.9 [range: 145.0 to 181.0] cm) who underwent dynamic CT of the upper abdomen at hospital B (blinded for review) between September 2020 and January 2021 were randomly selected. The height and BW of these patients were obtained from a questionnaire, as they could not be measured on the same day as the CT scan. Cases weighing 76 kg or more were excluded from the study because they could not maintain 600 mgI/kg and were fixed at 150 mL at 300 mgI/mL.

### CT acquisition

CT scans for lung cancer screening in Group A and B were performed using an Aquilion Prime SP (Canon Medical Systems, Ohtawara, Japan). The parameters of the chest CT scan were as follows: tube voltage: 120 kVp; modulated tube current obtained by automatic exposure control (standard deviation [SD]: 35; slice thickness: 5 mm; reconstruction kernel: FC03; and tube current range: 10–100 mA); reconstruction kernels: FC03; standard adaptive iterative dose reduction using three-dimensional (AIDR 3D) processing; collimation: 1.0 mm × 40 rows; pitch factor: 1.475; and slice thickness: 5 mm. In all cases, the field of view was set larger than the subject size.

Dynamic CT scans of the upper abdomen for Group C were performed using the Aquilion Prime Beyond Edition (Canon Medical Systems, Ohtawara, Japan). The dynamic CT scan parameters of the upper abdomen were as follows: tube voltage: 120 kVp; modulated tube current obtained by automatic exposure control (SD: 9; slice thickness: 5 mm; reconstruction kernel: FC03; and tube current range: 10–600 mA); reconstruction kernels: FC03; mild AIDR 3D processing; collimation: 1.0 mm × 40 rows; pitch factor: 0.825; and slice thickness: 5 mm.

The CT protocol for the upper abdominal dynamic CT used bolus tracking method, 15 s after contrast enhancement reached 150 HU in the region of interest (ROI) within the abdominal aorta for the upper abdominal arterial phase and 20 s after the arterial phase for the portal phase, with a delayed phase of 150 s after contrast injection. The BW reported by the patient in the iodine contrast questionnaire was used. The iodine contrast enhancement dose was set at 600 mgI/kg of total iodine. The iodine contrast media used were iohexol 300 mgI/mL (Omnipaque, GE HealthCare Pharma, Tokyo, Japan), ioversol 320 mgI/mL (Optiray, Guerbet Japan, Tokyo, Japan), and iomeprol 350 mgI/mL (Iomeron, Bracco Japan, Tokyo Japan). The iodine contrast was injected over 30 s using a dual-head power injector (DualShot, Nemoto Kyorindo, Tokyo, Japan). In all examinations in this study, breath holding was performed during inspiration during CT scanning.

### Optimal slice position for D_eff_ measurement method in group A and B

**Fig. 1:**
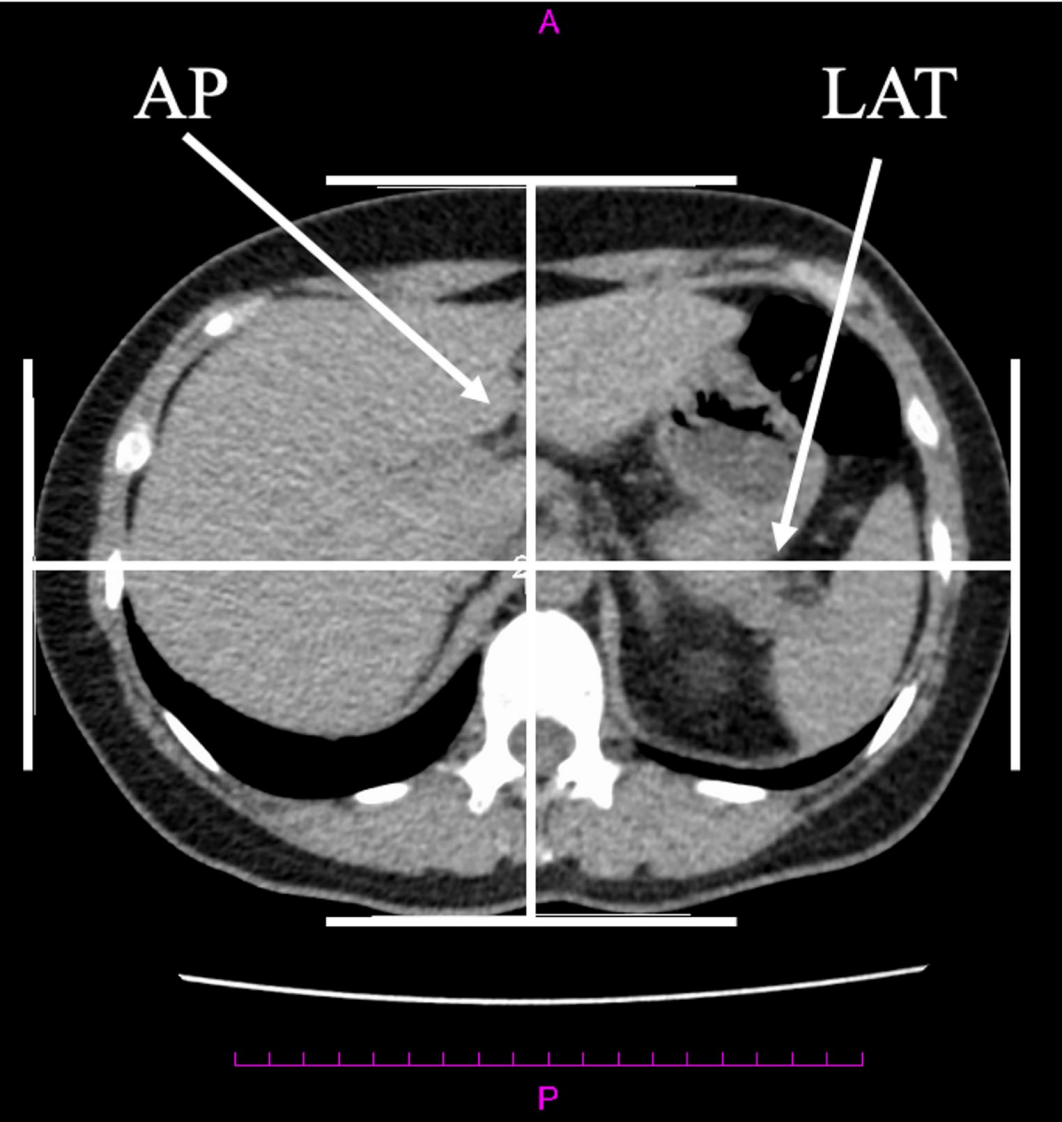
The method of measuring anterior-posterior (AP) and lateral (LAT) diameters in axial imaging. The D_eff_ was calculated from the AP and LAT diameters by radiological technologists. $\:{D}_{eff\_axial}\:=\sqrt{AP\:\times\:\:LAT}$. *D*_*eff*_ effective diameter, *D*_*eff_axial*_ effective diameter value measured from the axial image

**Fig. 2:**
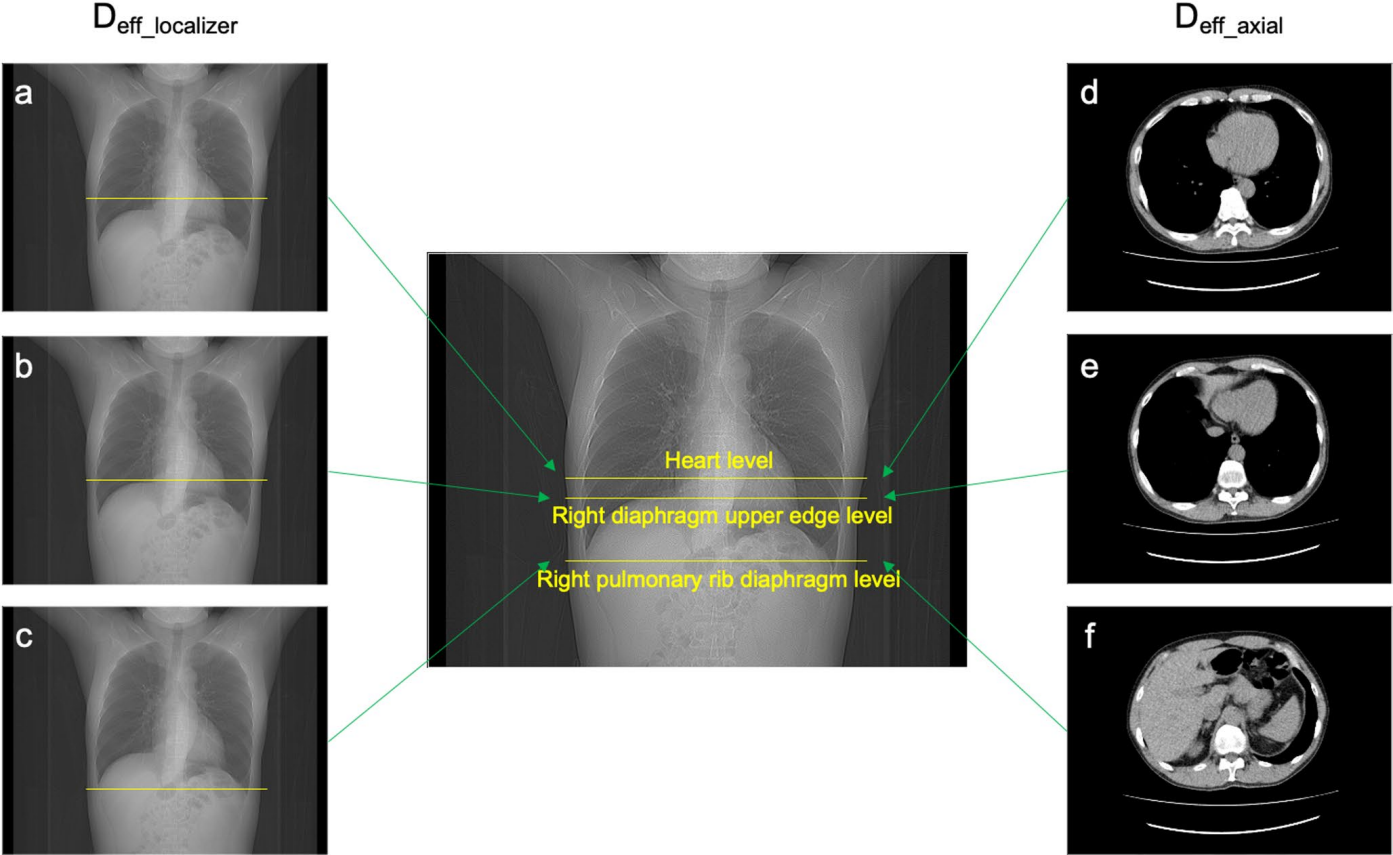
The D_eff_ at the heart, diaphragm upper edge, and right pulmonary rib diaphragm levels were measured in the localizer radiograph and axial images. The D_eff_localizer_ values measured from the localizer radiograph were transferred from the lateral diameter at the (**a**) heart, (**b**) right diaphragm upper edge, and (**c**) right pulmonary rib diaphragm levels. The D_eff_axial_ values measured from the axial images were transferred from the anterior-posterior and lateral diameters at the (**d**) heart, (**e**) right diaphragm upper edge, and (**f**) right pulmonary rib diaphragm levels. *D*_*eff*_ effective diameter, *D*_*eff_localizer*_ effective diameter value measured from the localizer radiograph, *D*_*eff_axial*_ effective diameter value measured from the axial image

D_eff_ was measured using the image measurement function ShadeQuest/ViewR (FUJIFILM, Tokyo, Japan) at the heart, right diaphragm upper edge, and right pulmonary rib diaphragm levels in the localizer radiograph and axial images (Figs. 1 and 2). The D_eff_ in Group A was measured by a radiological technologist (14 years of experience, certification in X-ray CT technology). The D_eff_ for the inter-rater reliability in Group B was measured by five radiological technologists (years of experience (2 years; 2 people, 11 years; 1 person, 20 + years; 2 people), certification in X-ray CT technology; 2 people). All measurers were trained in advance using 10 cases. The measurement positions were three cross sections near the diaphragm that were considered easy to select for chest, abdominal, and chest-to-pelvis CT scans. The D_eff_ values measured from the localizer radiograph (D_eff_localizer_) were transferred from the lateral (LAT) diameter using Eq. 1 [8]. This Eq. 1 an established formula from the literature 8.

1 $$\:\begin{aligned}{D}_{eff\_localizer}&=\:5.899298\:+\:0.3270494\:\times\:\:LAT\\&+\:0.009978896\:\times\:\:{LAT}^{2}\end{aligned}$$

The D_eff_ values measured from the axial image (D_eff_axial_) were calculated from the anterior-posterior (AP) and LAT diameters using Eq. 2 [8].

2 $$\:\begin{array}{c}{D}_{eff\_axial}\:=\sqrt{AP\:\times\:\:LAT}\end{array}$$

### Relationship between D_eff_localizer_ and D_eff_axial_ and BW in group A

Correlation coefficients between D_eff_localizer_, D_eff_axial_, and BW were determined for each cross-section. A linear regression equation was obtained using the measurement method and slice section with the highest correlation coefficient. The estimated BW was calculated using a linear regression equation:

3 $$\:BW\left[kg\right]=a\:\times\:\:{D}_{eff}\left[cm\right]+b,$$

where a and b are the regression coefficients.

This optimized regression model was subsequently used to estimate BW for Group C.

### Effect of iodine contrast in group C

**Fig. 3:**
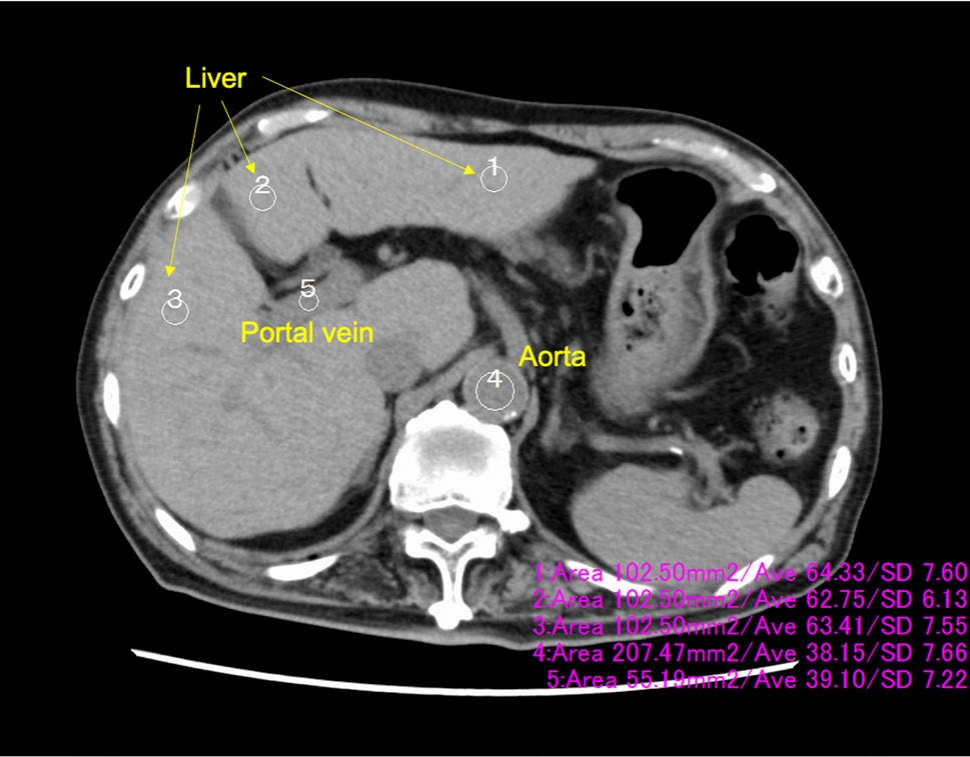
The region of interest was placed in a portal vein inflow slice. The CT values and standard deviation were measured in the liver, aorta, and portal vein

CT values and SD of CT values in ROI of the liver, aorta, and portal vein were measured in a portal vein inflow slice. The CT value in the liver was measured using a 100 mm^2^ ROI and averaged over three ROIs. The CT values of the aorta and portal vein were measured in ROI size of 200 and 50 mm^2^, respectively (Fig. 3). The increased liver and portal vein CT values in the portal phase were calculated by subtracting the corresponding values in the non-contrast-enhanced CT phase. Similarly, the increased aorta CT values in the arterial phase were obtained by subtracting the non-contrast-enhanced CT phase.

CT enhancement based on estimated BW was simulated based on the iodine amount (HU/gI) calculated for CT value enhancement of the aorta in the arterial phase, the portal vein and liver in the portal phase.

### Estimated BW error in group C

We analyzed the reported and estimated weights in Group C using the Brand–Altman method [9]. In addition, we calculated the mean absolute error (MAE) between the estimated BW derived from D_eff_axial_, and the reported BW of patients undergoing dynamic CT of the upper abdomen. The MAE was calculated using the following equation:

4 $$\:MAE=\frac{1}{N}\sum\:_{i=1}^{N}\left|{y}_{i}-{\widehat{y}}_{i}\right|\:$$

where, N is the total number of subjects, y_i_ is the reported BW, and ^y_i_ is the estimated BW.

### Statistical analysis

A normality test was performed using the Shapiro-Wilk test. Correlation analysis was performed by Pearson’s product-moment correlation (r), Spearman’s correlation coefficient (r_S_) and paired t-test using statistical software (EZR; Saitama Medical Center, Jichi Medical University, Saitama, Japan) [10]. A paired t-test was performed in Group C to assess whether significant differences existed between actual CT values and those simulated using estimated BW. Inter-rater reliability was assessed using the cross-sectional ICC, calculated with IBM SPSS Statistics version 20 (IBM, Armonk, NY, USA). The ICC ranged from − 1 to + 1. ICC values were interpreted as follows: 0.40, poor to fair agreement; 0.41–0.60, moderate agreement; 0.61–0.80, substantial agreement; and 0.81–1.00, almost perfect agreement [11].

## Results

### Relationship between D_eff_localizer_, D_eff_axial_ and BW in group A

**Fig. 4:**
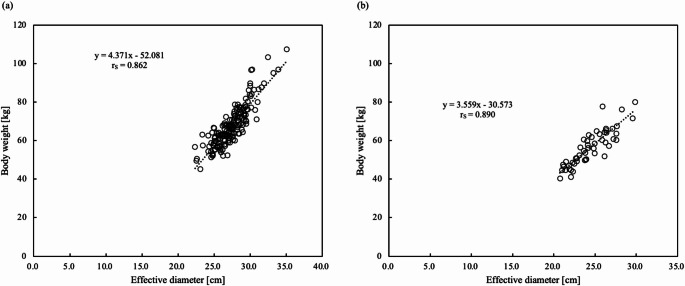
Scatter plot of the D_eff_axial_ relative to BW. (**a**) The D_eff_axial_ at the right diaphragm upper edge level showed the highest correlation in men. (**b**) The D_eff_axial_ at the right pulmonary rib diaphragm level showed the highest correlation in women. *D*_*eff_axial*_ effective diameter value measured from the axial image, *BW* body weight

The relationships between D_eff_localizer_ and D_eff_axial_ and BW are shown in Table 1. D_eff_axial_ and BW showed the highest correlation (r_S_ = 0.862) at the right diaphragm upper edge level in axial imaging for men (Fig. 4a). The D_eff_axial_ at the right pulmonary rib diaphragm level showed the highest correlation (r_S_ = 0.890) for women (Fig. 4b).

**Table 1:** The correlation coefficient between D_eff_ and BW, and linear regression equations

Measurement method	Slice level	Gender	*r*_S_	*p* value	a	b
D_eff_localizer_	Heart level	Men	0.793	< 0.01	3.395	−28.779
		Women	0.827	< 0.01	2.499	−8.120
	Right diaphragm upper edge level	Men	0.801	< 0.01	3.408	−27.781
		Women	0.809	< 0.01	2.847	−15.298
	Right pulmonary rib diaphragm level	Men	0.787	< 0.01	3.512	−29.141
		Women	0.881	< 0.01	3.611	−32.315
D_eff_axial_	Heart level	Men	0.855	< 0.01	4.478	−55.678
		Women	0.874	< 0.01	3.479	−30.554
	Right diaphragm upper edge level	Men	0.862	< 0.01	4.371	−52.081
		Women	0.871	< 0.01	3.535	−30.906
	Right pulmonary rib diaphragm level	Men	0.857	< 0.01	4.065	−42.796
		Women	0.890	< 0.01	3.559	−30.573

*D*_*eff*_ effective diameter, *BW* body weight, *r* correlation coefficient, *D*_*eff_localizer*_ effective diameter value measured from the localizer radiograph, *D*_*eff_axial*_ effective diameter value measured from the axial image;

a and b, regression coefficients

Estimated BW in Sect. 3.3 was calculated using the regression equation based on D_eff_ at the right diaphragm upper edge level for men and at the right pulmonary rib diaphragm level for women, as these groups exhibited the highest correlation coefficients.

### Inter-rater reliability in group B

The ICC (2,1) values for men and women are shown in Table 2. The ICC (2,1) values ranged from 0.860 to 0.992. The inter-rater reliability was almost perfect for all measurements of D_eff_localizer_ and D_eff_axial_.

**Table 2:** The ICC for inter-rater reliability. The inter-rater reliability was almost perfect for all of D_eff_localizer_ and D_eff_axial_ measurements (ICC [1,2] = 0.860–0.992)

Measurement method	Slice level	Gender	ICC(2,1)	95% CI	
D_eff_localizer_	Heart level	men	0.940	0.949	0.980
		women	0.872	0.861	0.939
	Right diaphragm upper edge level	men	0.860	0.805	0.917
		women	0.924	0.887	0.951
	Right pulmonary rib diaphragm level	men	0.944	0.938	0.975
		women	0.934	0.899	0.957
D_eff_axial_	Heart level	men	0.979	0.973	0.990
		women	0.936	0.917	0.965
	Right diaphragm upper edge level	men	0.992	0.991	0.997
		women	0.932	0.898	0.956
	Right pulmonary rib diaphragm level	men	0.981	0.979	0.992
		women	0.958	0.937	0.973

*ICC* intraclass correlation coefficient, *95% CI* 95% confidence interval, *D*_*eff_localizer*_ effective diameter value measured from the localizer radiograph, *D*_*eff_axial*_ effective diameter value measured from the axial image

### Effect of iodine contrast in group C

**Fig. 5:**
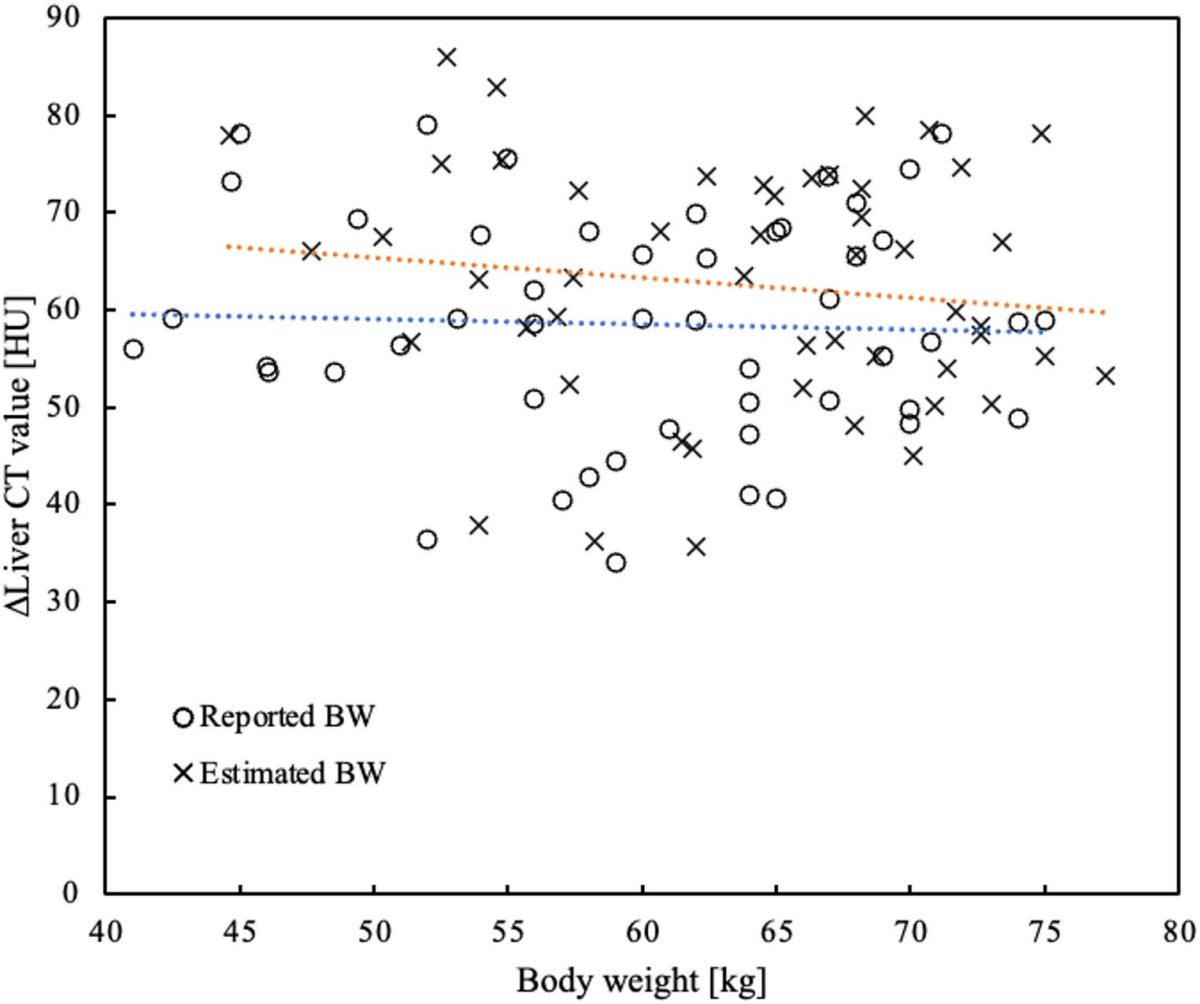
Scatter plot of BW relative to the increased liver CT values in the portal phase. The mean value of the actual increased CT values was 58.5 HU (± 11.5 HU) for reported BW (blue approximation curve). The mean value of the simulated CT value was 62.5 HU (± 12.3 HU) for estimated BW (orange approximation curve). The simulated CT values were significantly higher than the actual increased CT values (*p* < 0.001). *BW* body weight, *CT* computed tomography, *HU* Hounsfield unit

Figure 5 shows a scatter plot of increased CT values in the liver relative to BW. The simulated CT values corresponding to the estimated BW were significantly higher than the actual increased CT values for the reported BW (*p* < 0.001). The mean increase in CT values was 58.5 HU (± 11.5 HU).

**Fig. 6:**
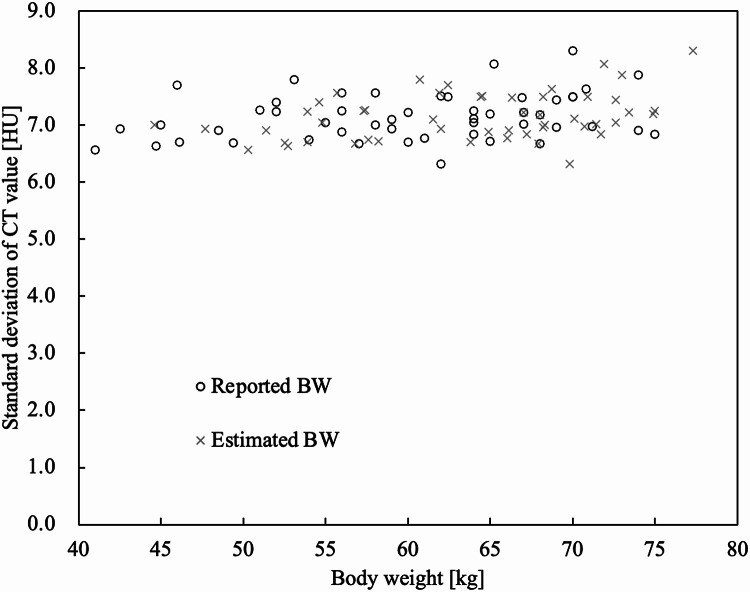
Scatter plot of BW relative to the standard deviation of liver CT value. The SD of liver CT values was plotted across equivalent distribution from low to high reported and estimated BW. *BW* body weight, *CT* computed tomography, *SD* standard deviation

Figure 6 shows the SD of the CT values of the relative BW of the liver in the non-contrast-enhanced CT. The SD values were plotted with an equivalent distribution from low to high BW. The mean SD value was 7.1 HU.

**Fig. 7:**
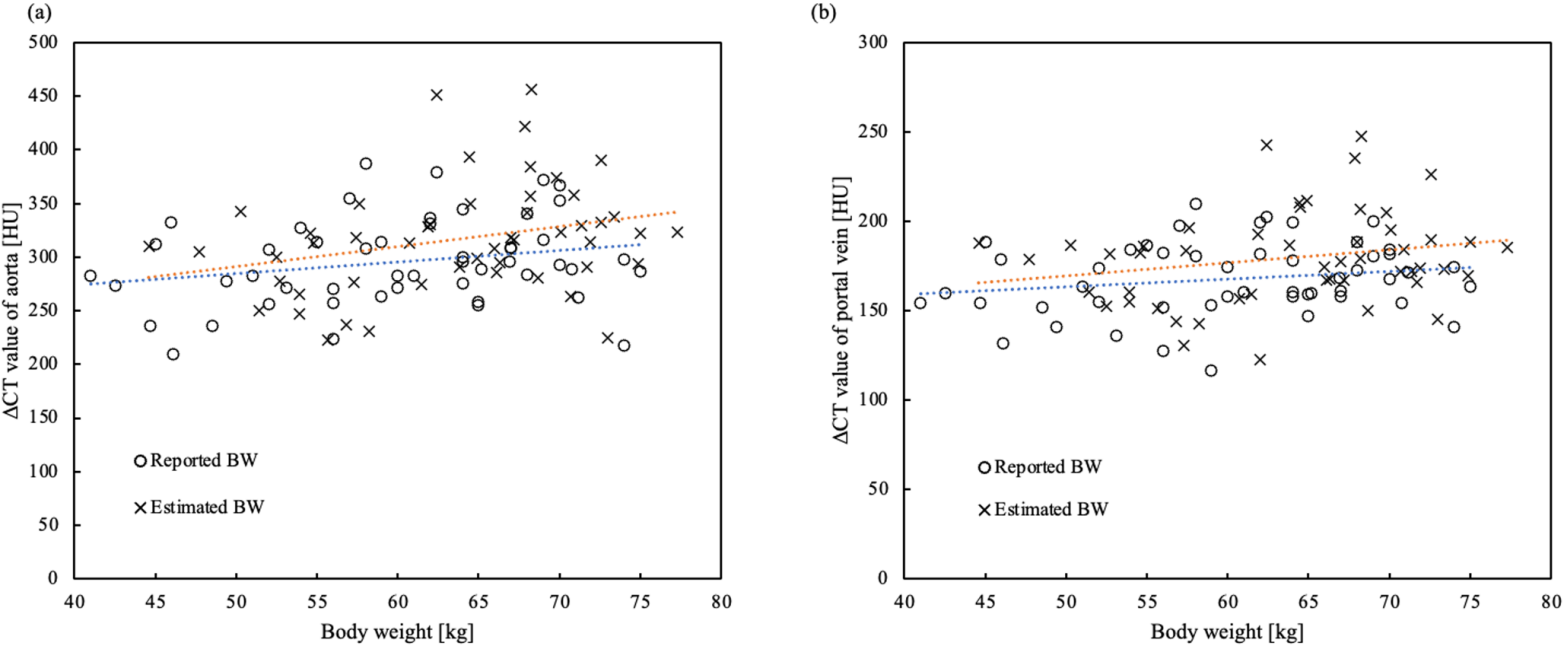
Scatter plots showing BW relative to CT values for (**a**) the aorta and (**b**) the portal vein. The blue line represents the regression line for the actual CT values, while the orange line represents the regression line for the simulated CT values. For both (**a**) the aorta and (**b**) the portal vein, the simulated CT values were significantly higher than the actual CT values (*p* < 0.001). *BW* body weight, *CT* computed tomography

Figure 7a shows a scatter plot of the CT values of the aorta during the arterial phase. Figure 7b shows a scatter plot of the CT values of the portal vein in the portal phase. For both the aorta and the portal vein, the simulated CT values were significantly higher than the actual CT values (*p* < 0.001).

### Estimated BW error

**Fig. 8:**
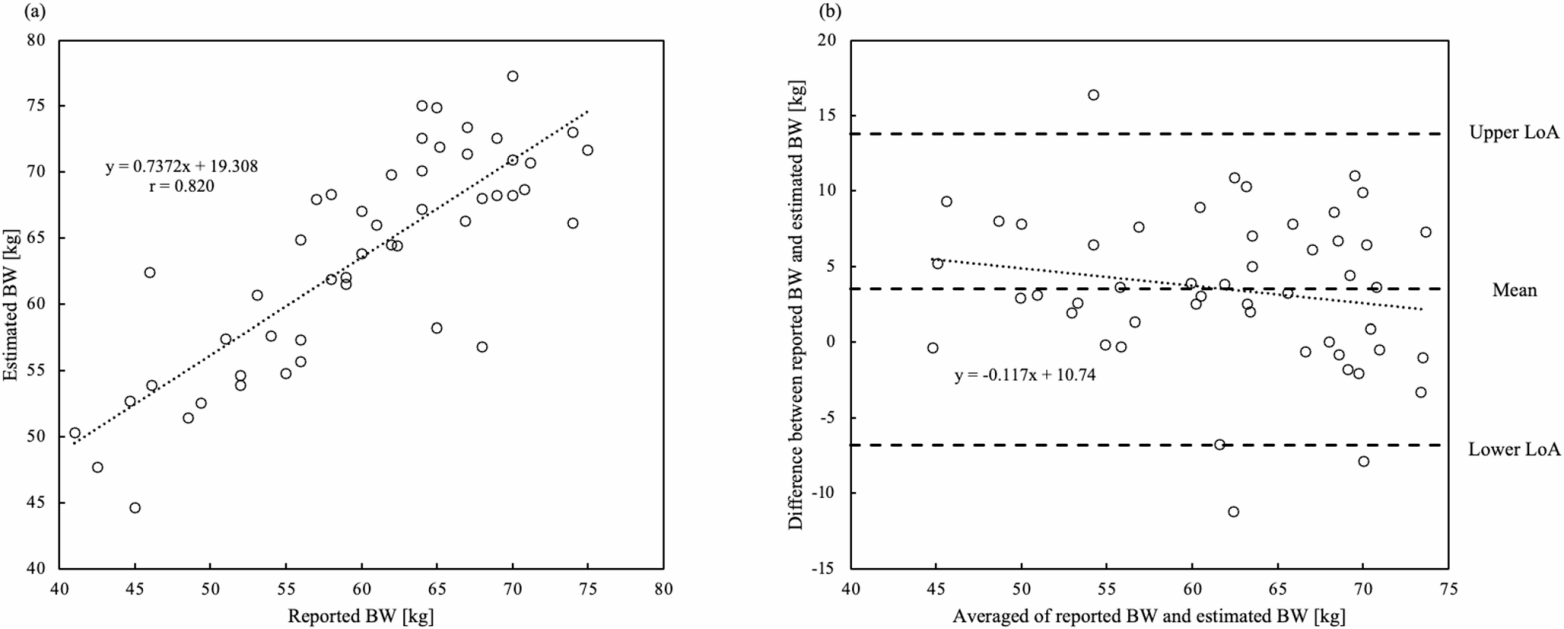
(**a**) Scatter plot of reported BW relative to estimated BW. The reported BW and estimated BW showed a high correlation (*r* = 0.820). (**b**) A Bland–Altman plot comparing reported and estimated BW in 50 patients undergoing dynamic CT of the upper abdomen. No significant proportional error was observed (*p* = 0.202). *BW* body weight, *SD* standard deviation, *CT* computed tomography, *LoA* limits of agreement

Figure 8 shows the scatter plot of reported BW relative to estimated BW and a Bland–Altman plot of reported and estimated BW. The estimated BW was within ± 5 kg for 54% of patients and within ± 10 kg for 90%. The MAE between the estimated and reported BW was 3.5 kg.

## Discussion

The measurement positions were three cross-sectional levels near the diaphragm that were considered easy to select in chest, abdominal, and chest-to-pelvis CT. D_eff_ values were highly correlated with BW (r_S_ = 0.787–0.890). (Table 1; Fig. 4). The highest correlation was observed in men at the right diaphragm upper edge level on axial images (r_S_ = 0.862), and in women at the right pulmonary rib diaphragm level on the axial images (r_S_ = 0.890). The inter-rater reliability was almost perfect (ICC [1,2] = 0.860–0.992) (Table 2). The three measurement levels near the diaphragm correspond to the upper end of the scan range for non-contrast-enhanced CT of the upper abdomen, thereby eliminating the need for an additional axial image to measure D_eff_axial_. D_eff_ has been widely reported to correlate strongly with BW [6]. Although the value of D_eff_ varies by cross-sectional level, it consistently shows a strong correlation with BW across different measurement points. In either case, BW can be estimated using the linear regression equations presented in Table 1.

In this study, D_eff_ was calculated at cross-sectional levels near the diaphragm, showing the highest correlation with BW at the right diaphragm upper edge level for men and at the right pulmonary rib diaphragm level for women. D_eff_ demonstrated a strong correlation with BW in the chest than in the abdomen [6, 12, 13]. In a study estimating BW from images using deep learning, the MAE was lower in the chest than in the abdomen [5], and a heatmap analysis showed an emphasis on the diaphragmatic region to predict BW [7]. These findings support estimating D_eff_ at the right diaphragm upper edge level for men and at the right pulmonary rib diaphragm level for women.

The mean increase in actual CT values was 58.5 HU (± 11.5 HU) for reported BW. The mean value of the simulated CT value was 62.5 HU (± 12.3 HU) for estimated BW (Fig. 5). In this phase, hepatic enhancement has been reported to require at least 50 HU [1]. We believe that the estimated BW using D_eff_ can meet this requirement. Future studies should verify the estimation of iodine contrast doses based on BW as determined by D_eff_. The SD of liver CT values was distributed evenly across the range of BW (Fig. 6), indicating that image noise remained consistent regardless of BW, likely due to automatic exposure control. The simulated CT values of the aorta in arterial phase and of the portal vein in the portal vein phase were significantly higher than the actual CT values (*p* < 0.001) (Fig. 7). Consequently, using estimated BW derived from D_eff_ may result in systematic overestimation of CT values, particularly in highly vascular structures such as the aorta and portal vein during contrast-enhanced phases of dynamic CT of the upper abdomen.

The MAE between the estimated and reported BW was 3.5 kg. In comparison, a deep learning method using CT scout images achieved an MAE of 2.75 kg for a chest dataset [5]. This difference corresponds to 2.1 gI in iodine contrast dose. We believe that such a difference has minimal impact on renal function and image quality.

Several methods for estimating BW from X-ray or CT images have been reported [4, 5, 7]. Deep learning methods require CT images to be loaded into specialized software. In contrast, our technique only requires measuring AP and LAT diameters from axial images on the CT console, making it easy to implement in any hospital. D_eff_localizer_ may affect measurement accuracy when the patient is not positioned at the center of the gantry. When the patient is farther away from the tube relative to the distance from the tube to isocenter, the patient’s image is minified [14]. Conversely, when the patient is closer to the tube compared to the distance to isocenter, the patient’s shadow is magnified [14]. D_eff_axial_ requires that the entire subject be included in the image to accurately measure both the AP and lateral diameters. This requires that a full FOV image encompassing the entire patient cross-section be reconstructed [14].

A major limitation of this study is that it was not conducted prospectively using iodine contrast doses determined by estimated BW from D_eff_. The applicable BW range for estimation is from 40 to 75 kg. The possibility of variation in measurement levels due to respiration. That this study is based on a specific protocol for upper abdominal CT only.

## Conclusions

This study validated the measurement accuracy of using D_eff_axial_ to determine contrast dose in patients with unknown BW. Inter-rater reliability was almost perfect for all D_eff_ measurements. BW estimated from D_eff_ showed a strong correlation with measured BW and may serve as a practical alternative in cases where BW is unknown. However, contrast dosing based on estimated BW was not implemented in this study, and further quantitative evaluation of its clinical impact is needed.

## Data Availability

The data that support the findings of this study are not openly available due to ethical restrictions and are available from the corresponding author, upon reasonable request.
